# Development and Evaluation of Acceptability and Feasibility of a Web-Based Intervention for Patients With Bipolar Disorder in Iran: Implementation Study

**DOI:** 10.2196/23360

**Published:** 2021-08-17

**Authors:** Agaah Ashrafi, Maryam Tabatabaee, Vandad Sharifi

**Affiliations:** 1 Department of Psychiatry, Roozbeh Hospital Tehran University of Medical Sciences Tehran Iran

**Keywords:** bipolar disorder, psychoeducation, web-based intervention, feasibility, acceptability

## Abstract

**Background:**

Psychoeducation for bipolar disorder has a significant impact on symptoms and treatment adherence. In Iran, as a low-resource setting, infrastructural barriers, such as inadequate mental health professionals, difficulties in transportation, and costs of care, may hinder optimum delivery of this evidence-based intervention to patients.

**Objective:**

This study sought to explore the acceptability and feasibility of a web-based intervention for bipolar patients in Iran.

**Methods:**

A website has been developed as a platform for providing psychoeducational content about bipolar disorder. Patients were chosen via a convenient sampling method in 2018-2019. The main component of the intervention included streaming 7 weekly video clips after attending a single in-person meeting, as well as a medication self-monitoring application. Information was collected about the feasibility and acceptability of the intervention.

**Results:**

We invited 45 patients from the day center and the outpatient clinic of Roozbeh psychiatric hospital and some private clinics in Tehran. Of the 23 patients (51%) who attended the first in-person session and provided informed consent, 14 patients dropped out during the study. While 9 patients completed the course (attended 4 or more online sessions), only 5 watched all the video sessions. The rate of adherence to the intervention and frequency of exposure to the website were much higher for those recruited from the private and outpatient clinics.

**Conclusions:**

This web-based intervention can be feasible and acceptable only for a subgroup of patients with specific educational status and socioeconomic level.

## Introduction

Bipolar disorder (BD) is a substantial public health problem, with lifetime prevalence of around 2.4% in the general population according to world mental health survey in 2011 [1] and 1% among the Iranian population [2]. Patients frequently present relapsing episodes and usually undergo subsyndromal symptoms, cognitive problems, functional impairment, and repeated hospitalizations which cause significant burden both for their families and the whole community. Medications is considered as a gold-standard treatment modality, however, psychosocial interventions, such as psychoeducation (PE), self-help, and psychotherapy (individual, couple, and family), have shown promising treatment outcomes for BD [3].

PE is considered an effective modality in the treatment and management of BD [4-6]. In a recent systematic review conducted by Demissie et al [5] in 2018, it was concluded that PE would enhance patients’ adherence to treatment, knowledge and attitude toward the condition and their quality of life as well as it would minimize relapse rates and hospitalizations. In Iran, several studies which investigated the effectiveness of PE interventions showed that PE significantly decrease relapse and re-hospitalization rate [7]. Faridhosseini and colleagues (2017), investigated the effectiveness of a culturally adapted structured PE program for BD. They also concluded that PE for patients with BD could improve their quality of life and minimize relapse risk [8].

In contrast to the vast literature regarding the efficacy of PE programs, there are still some limitations in the application and delivery of these services, specifically in low resource settings such as Iran. Traditionally, these services are provided face-to-face, which is not always possible especially for patients who have low treatment commitments or major problems for attending the face-to-face sessions, including long distance, limitation in affording needed time, energy and/or money, and limited access to health professionals.

Novel web-based technologies offer an opportunity to employ standardized psychological treatments that can deal with some of the aforementioned limitations. Considering the fact that these technologies are widely accessible 24-hours a day, the timing of interventions can be tailored to the patients’ needs and availability [9].

In some high-income countries such as the United Kingdom, health-care policymakers have shown interests about employing the power of the internet to allow patients to take more responsibility in their illness management. “Beating Bipolar” is a web-based PE intervention developed in the United Kingdom and has proven to be as a promising PE treatment modality for BD patients; this intervention focuses on illness awareness, adherence to treatment, early detection of recurrence and lifestyle regularity [9-11].

Taken together, to fill the gap between availability and demand, we need to think of a treatment platform which is more accessible and feasible. In developing countries such as Iran, the obstacles, including distance, and limited number of mental-health staffs in remote areas, have posed serious problems regarding management of this group of patients and this calls for employing novel treatment approaches such as online PE. Here we tend to offer a preliminary web-based platform for PE and to evaluate its acceptability and feasibility for a group of patients with BD in Iran.

## Methods

### Developing the Web-Based Intervention

After reviewing available widely used and studied web-based intervention platforms in developed countries [9,12-14], we developed a preliminary design and proposed it to a professional website developer firm in Tehran. The website included 2 main parts: (1) 7 visually animated video clips (modules) with PE-related content that were simply narrated and had a maximum length of 20 minutes; the content was based on evidence-based knowledge on BD and our team’s previous experience with face-to face PE programs [15]; (2) weekly medication tables for patients to self-record their medications to improve their feelings of agency and mastery and emphasize the importance of self-monitoring and adherence to medications. It is worth mentioning that to respect the privacy of patients, each user was allocated a personal portal to register their progress and data.

Following the first face-to-face session, the 7 internet-based modules were provided. Table 1 shows the topics that were covered.

**Table 1:** Contents of the web-based psychoeducation course for bipolar disorder.

Session/week	Content
1	In-person orientation meeting
2	Bipolar disorder and definitions
3	Etiology and risk factors
4	Bipolar disorder, medications and treatments
5	Alarm signs and relapse prevention
6	Adaptation to bipolar disorder
7	Problem solving, an essential skill for patients
8	Other topics such as marriage, exercise, driving, diet, occupation, and more for bipolar patients and summary

Topics, as mentioned in Table 1, were incorporated into the visual content (educational video clips) and written material (downloadable pdf files). To persuade and encourage patients to use the website during the course, we sent them a reminder text message at the beginning of each week. Moreover, a module to record questions from patients was incorporated in the website, and it was answered regularly by one of the authors. After developing a preliminary version, we randomly chose 3 patients of different ages and genders to participate and give us some feedback regarding their experience in a pilot course using this website.

### Implementation and Evaluation of the Web-Based Intervention

#### Participants

We recruited subjects from the day center and outpatient clinic of Roozbeh Psychiatric Hospital and some private clinics in Tehran. Our inclusion criteria were patients with a DSM-5 BD, diagnosed by a psychiatrist, age 15-65 years old, being able to access a computer and the internet, a minimum educational level of the 6th grade in primary school, no previously receipt or completion of any PE courses, and full or partial remission at the time of the study as indicated by their psychiatrist. We excluded patients with severe visual impairment, intellectual disabilities, or neurodevelopmental disorders.

#### Procedures

**Figure 1:**
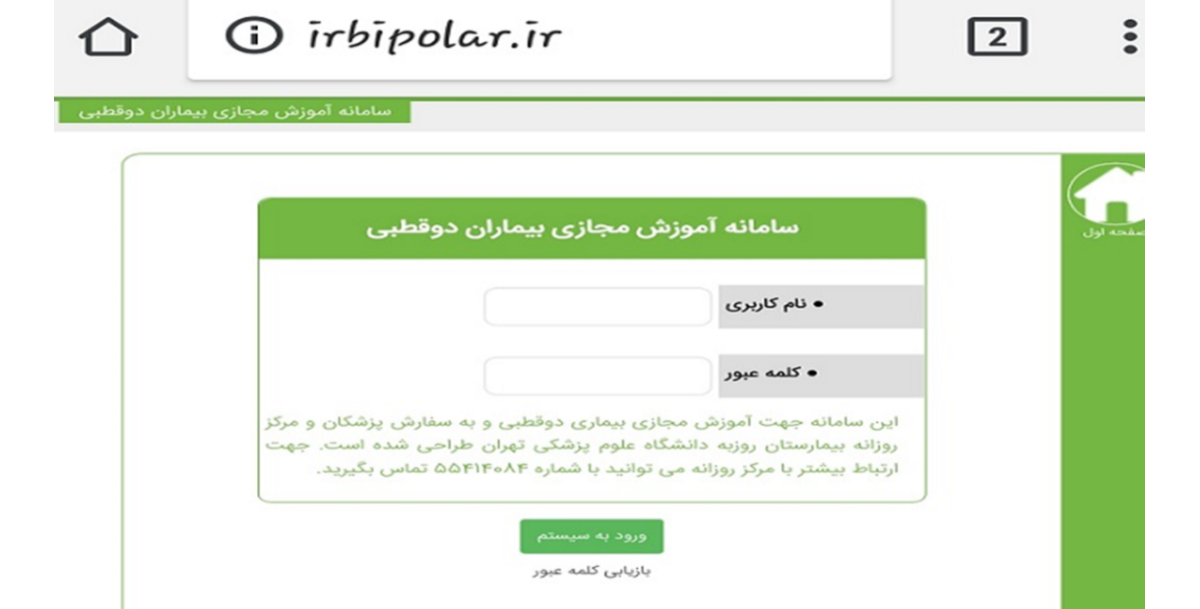
The front page of the irbiplar.ir website.

The first session of each course (1st module) was held in person to familiarize each patient with other participants, the research team, and the overall process of the project and to obtain informed consent. Patients were informed regarding the importance of PE in the management of psychiatric conditions and more specifically BD. In each round of the interventions, we invited patients via a phone call, and we reminded them of the session time via text message 1 day before the meeting. In this first session, personal username and password were allocated to each patient to fill in their data. After this session, we invited the patients to participate in our study for 7 consecutive weeks and to expose themselves to the aforementioned PE video clips on the website. Figure 1 demonstrates the front page of the website where the patients were requested to enter their own username and password to access their individual profile. The Farsi language was selected for the website so a language barrier would not become an issue for our patients who all were Iranian.

**Figure 2:**
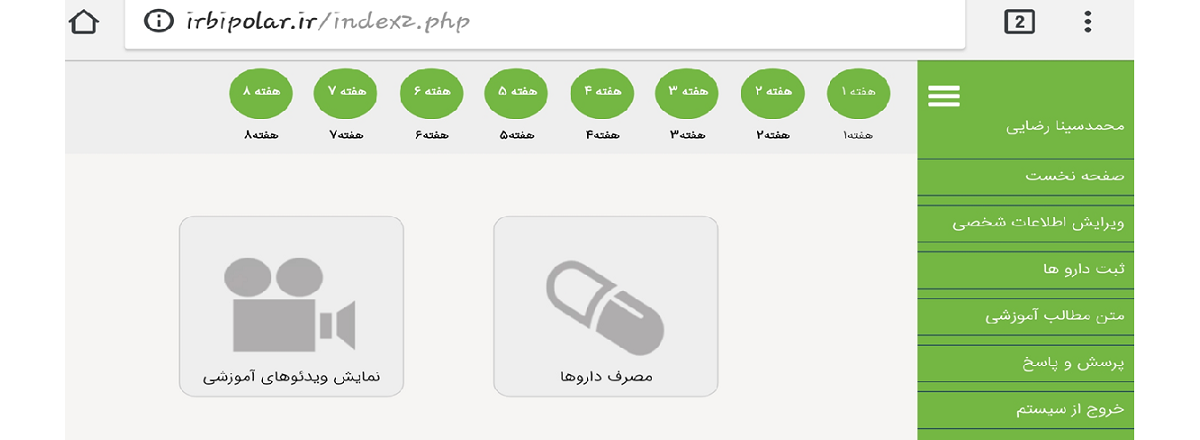
The main page of the irbipolar.ir website.

Figure 2 shows the main page of the website, which showed 8 round circles at the top of the page as a manifestation of the course progress and the week the patients were in at any time, 2 main sections in the middle of the page, and some ancillary tabs on the right side.

**Figure 3:**
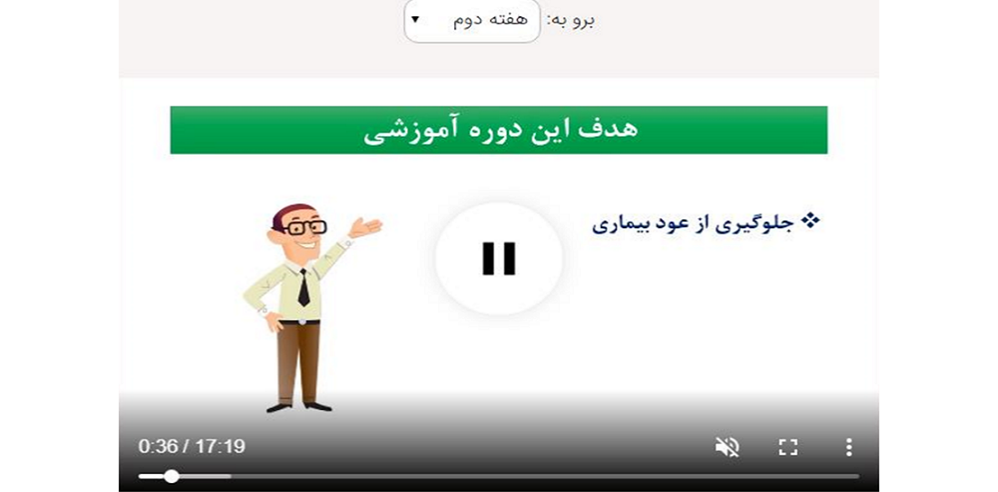
Page with the psychoeducation video clips.

**Figure 4:**
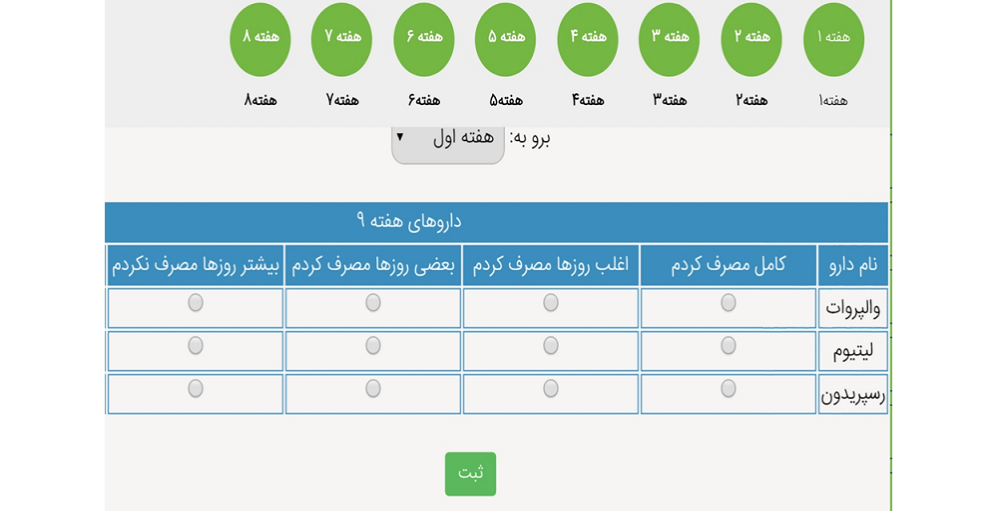
The weekly table for recording the pattern of adherence to medication.

One of the 2 main sections linked to the weekly streaming PE video clips (Figure 3; Multimedia Appendix 1), and the other section showed a weekly timetable of medications to take and allowed them to indicate their pattern of actual medication administration during the week (Figure 4).

Initially, this section was supposed to be a reminder to regulate the patients’ patterns of taking medication as well as give them a sense of mastery over their treatment plan; however, we thought a reminder might be negatively viewed by the patients as being controlled and might even decrease their cooperation. Therefore, this section remained optional, although strongly suggested, for the patients to use.

It is worth mentioning that during the intervention period, patients received other mental health services as well, which included medications and if indicated, psychotherapy and rehabilitation services provided by the hospital. For cases for whom we suspected a relapse, patients were referred for a medication review, and afterwards if the patient was still interested in participating in the study, we asked him or her to update his or her medication table on the website and continue the course. If the patient was not still interested, he or she was excluded from the study.

#### Assessments

Our primary objective was to determine the level of feasibility and acceptability of this intervention. Feasibility outcomes were assessed via the recruitment rate, adherence to the website training course protocol, participant drop-out rate (participants who did not attend ≥4 weeks during their 8-week course). In addition, we conducted qualitative interviews with the team members and patients at the end of study to discuss obstacles and facilitators of the implementation platform and how to address them. Acceptability was assessed via the frequency and pattern of website use by participants, which was designed to be reported as an Excel file by the programmer of the website, as well as patients’ satisfaction rate, including whether the program was user-friendly, comprehensibility of the content, usefulness of the content, and usefulness of reminders. These variables were rated on a Likert scale, ranging from 1 to 5, for each item by the participants in terms of an interview conducted by 1 of the authors. SPSS v20 and Excel 2016 were used for statistical calculations and analyses as needed.

### Ethical Considerations

A written informed consent form was completed by each patient. Patients were assured that the data are confidential. The current design was approved by the Research Ethics Committee of Tehran University of Medical Sciences (IR.TUMS.MEDICINE.REC.1397.579).

## Results

### Patient Recruitment Process

Among 70 bipolar patients referred to Roozbeh Psychiatric Hospital in the second half of 2018, 12 were eligible to be invited to our study. Of these, 2 attended the first in-person session, and 8 could not participate because they had already completed the same course in previous face-to-face sessions. The remaining 50 patients of the 70 patients referred were excluded for not meeting the inclusion criteria, such as not having enough interest or lack of familiarity with the internet. Then, we invited an additional list of 33 patients from the hospital outpatient clinics and private clinics in Tehran in January 2019.

**Figure 5:**
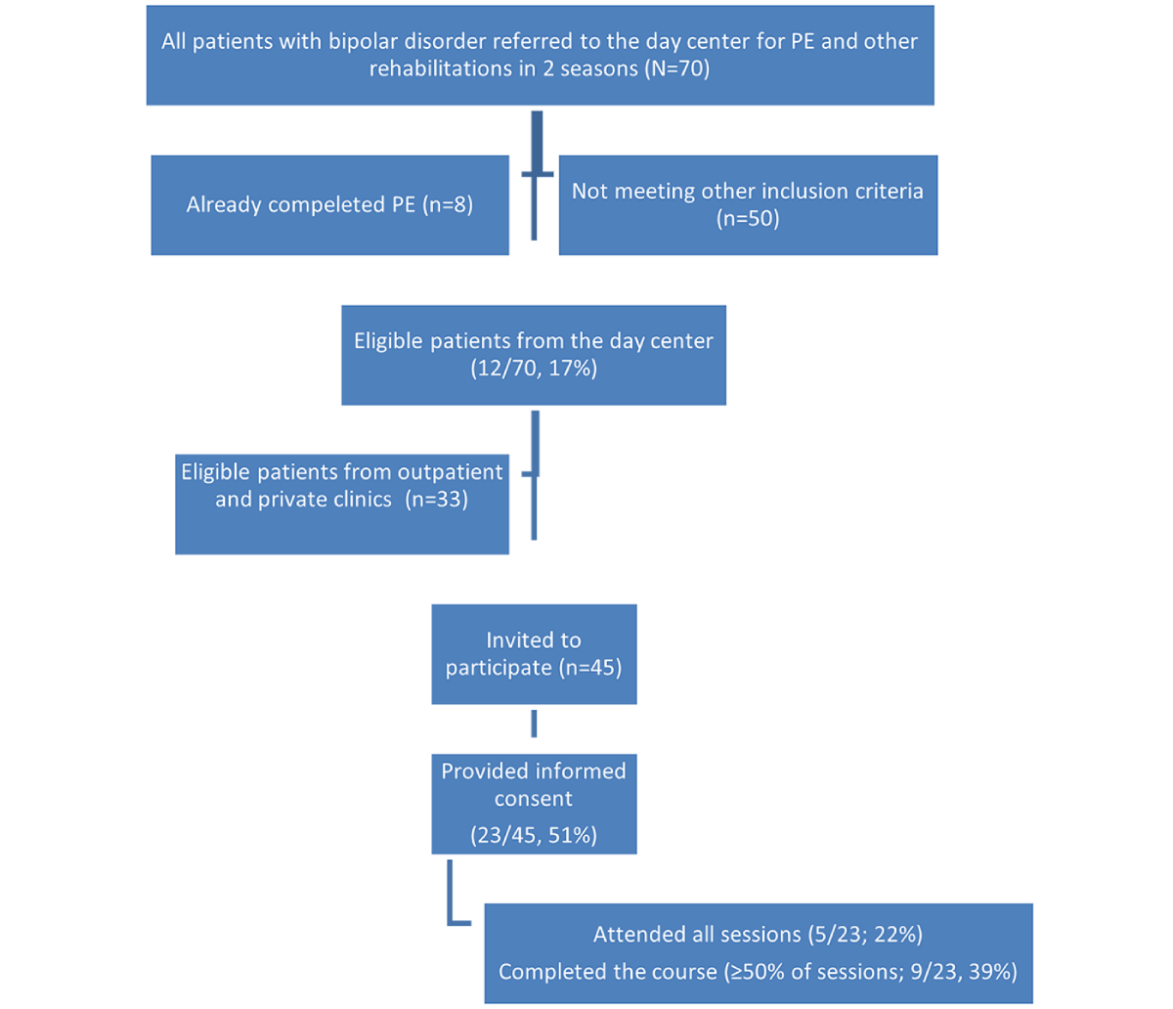
The flow of subjects in the study. PE: psychoeducation.

Finally, among the 45 invited patients, 23 attended the first in-person session (51%) and agreed to participate in the study. Figure 5 shows the sampling process.

### Feasibility Outcomes

#### Recruitment Rate

Among the patients referred to the day center, the recruitment rate was 3% (2/70). It was 63% (21/33) for the outpatient and private clinics.

#### Adherence Rate

We defined a dropout as being absent in 4 or more of the 8 sessions of the whole course. There was a 100% dropout rate during the first round of the study, which was held for the patients referred to the day center of Roozbeh Hospital, as none of the patients attended online meetings. Among all patients, 14 patients dropped out during the study. Of the 9 patients who completed the course, only 5 (22%) attended all the sessions, and 4 (17%) patients were absent in some sessions, but they completed the course. Furthermore, the level of commitment of all patients to participate in each week is illustrated in Table 2.

**Table 2:** Adherence to the web-based psychoeducation modules.

Module	Number of patients who completed the module
1	23
2	12
3	9
4	9
5	8
6	7
7	6
8	6

#### Dropout Rate

As mentioned, among 23 patients who entered the study, 14 dropped out, which accounts for 61% of the total sample who attended the first in-person meeting. Of these 14 participants, 50% (7/14) provided reasons that could be considered as a “lack of enough motivation.” Table 3 shows the possible reasons for nonadherence (dropout) that were obtained from interviews with the participants.

**Table 3:** Dropout reasons (n=14).

Dropout reasons	n (%)
Relapse	2 (14)
Difficulty using web-based modules	3 (21)
Not having access to a computer during certain weeks of the course	2 (14)
Other reasons^a^	7 (50)

^a^Other reasons include those that could be considered a lack of motivation such as a lack of spare time and forgetfulness.

### Acceptability Outcomes

#### Frequency and Pattern of Website Use

This includes the number of patient logins to the website and the entire time that was spent on the website by patients. As shown in Table 4, an average frequency of 1-2 logins per week, each lasting about 21 minutes, by patients who completed the course indicates the relative acceptability of this course, while for noncompleters, this variable is meaningfully lower (Table 4). While 14 (14/23, 60%) watched at least one PE video, only 5 (5/23, 22%) patients watched all of the videos.

**Table 4:** Frequency and pattern of participant use of the website over the entire 7 weeks of the study.

Parameter	Patients who dropped out (n=14)	Patients who completed the course (n=9)
Total logins, n	40	117
Average logins per participant, mean (SD)	2.85 (1.75)	13 (3.85)
Total duration (minutes)	233	1333
Average duration per participant (minutes), mean (SD)	16.65 (12.23)	148 (29.1)

#### Participants’ Experiences

To assess participants’ experiences using the online platform, we interviewed 13 participants who at least watched one PE video to gain answers to satisfaction questions, with responses rated from 1 to 5 on a Likert scale; the results are presented in Table 5. The average range of satisfaction with each item shows the relative acceptability of the intervention.

**Table 5:** Participants’ experiences using the website (score range: 1-5).

Characteristic	Rating, mean (SD)	Mean rating divided by the total possible score of 5, %
User-friendly	4.2 (1.12)	84
Simplicity and usefulness of the contents	4.5 (0.86)	90
Usefulness of the Q&A panel	3 (1.37)	60
Usefulness of reminders	4 (1.20)	80

## Discussion

Generally, our findings demonstrated this intervention was feasible and acceptable only for some groups of patients with specific educational and socioeconomic status who met the requirements of the procedure.

### Principal Findings

In recent years, novel communication and information platforms have represented a promising prospect to offer psychological intervention via web-based tools and to tackle some of the drawbacks of face-to-face sessions. In comparison with in-person sessions, they are readily accessible and minimize the delivery timeline. Furthermore, the timing of the therapy can be arranged according to the specific need and availability of each user.

In developed countries, telemedicine and more specifically, telepsychiatric platforms (websites, applications) are becoming increasingly used in the treatment plan of bipolar patients [16-18]. In Iran, however, as a low-resource setting, this area is still young, and we do not have a strong evidence base regarding the efficacy and feasibility of web-based interventions for the management of psychiatric conditions.

Currently, PE, as one of the most studied psychotherapy modalities, has been revealed as a practical approach to the management and treatment of BD [19-21]. Considering the interest of many patients in searching the internet for their conditions and treatments, the vast amount of invalid and low-quality content that is currently available could put them at higher risk. Hence, it appears that providing a platform with evidence-based materials supported by responsive, knowledgeable staff to provide answers to patients anytime from anywhere via the platform is necessary. Furthermore, the low number of mental health professionals in many remote geographical zones with an increasing number of patients calls for adopting a novel approach to address the specific needs of this patient group in our country.

According to the results of our study, we can conclude that our web-based PE intervention platform is not feasible and acceptable for patients who were referred to the day center of Roozbeh Psychiatric Hospital. We speculate that for this first group of patients who may have a more severe illness and poorer educational and socioeconomic background that makes it likely they are clients of a nonprivate university hospital, the poor attendance is due to a lack of motivation or knowledge about the importance of these interventions in the management of their disorder as well as lower skill level or access to these types of technology-based services. However, this platform was sufficiently accepted and feasibly implemented for another group of patients who were recruited from private clinics or outpatient clinics in Roozbeh Hospital who were already more actively seeking the needed care and had better socioeconomic backgrounds.

### Obstacles and Facilitators to Designing the Website

#### Influential Factors

After holding a number of sessions, the research team reached consensus about influential factors in creating the website and sorted these factors into 2 categories: factors related to the individuals involved in the study and factors related to the intervention.

#### Factors Related to the Individuals Involved in the Study

For the web design team, the main obstacle was that our research team did not have any previous experience in designing a website, so we had to outsource this stage to a reliable web design firm. Since the web design firm did not have any professional knowledge regarding our subject, we had to hold a number of sessions to align our expectations and knowledge with their experience in designing an online platform.

Regarding the research team, a limited number of professional staff and insufficient team members for implementing different stages of the study and follow-ups meant one person carried all these responsibilities, which could increase the number of errors.

For patients, first, we should state that a noticeable proportion of patients did not have enough motivation or skills to participate in the current study, and some patients thought that participating in this study may lead to a relapse of their symptoms. Several patients also preferred face-to-face sessions and did not identify enough with the proposition of online platforms.

#### Factors Related to the Intervention

First, we can point to the novel feature of this intervention in our setting, which can result in some trial and error. Furthermore, we faced some unfortunate incidences such as filtering the website in the middle of the study, which happened accidently by the government. The main limitation was the necessity to have 1 face-to-face session at the initiation of the study for patients. This obstacle manifested as the participation gap between the potential 45 volunteers at the preliminary invitation and the ~50% attendance (n=23) at the first in-patient session. Another limitation was specifying a 1-week period for each module; according to some patients, they had a problem attending some modules due to a lack of spare time, sickness, or travelling, and sometimes, they lost their motivation in continuing the course when they were absent for 1 module. Another issue was that watching a video clip or answering some questions did not take more than 1 or 2 hours per week, and this limited involvement during the week could lead to decreased motivation to continue participation. We could have tackled this issue by providing daily tasks for participants to complete and revive their motivation on a regular basis.

Our results are consistent with the outcome of the qualitative study conducted by Poole and colleagues [18]; they analyzed the feasibility, acceptability, and impact of an internet-based PE platform, “beating bipolar.” They found it feasible to deliver and acceptable for patients to use via a computer. This intervention had a satisfying impact on insight concerning illness, health behavior, personal habits, and positive attitude toward medication [18]. However, in their study, thematic analysis was employed to describe the participants’ experience. In the current study, we used a more quantitative approach. Hidalgo-Mazzei et al [12] designed a simple smartphone application to provide PE contents for patients with BD. Consistent with our results, this type of online intervention was an efficient approach to improve self-management by patients with BD. However, their small sample size (51 patients) made generalization difficult, as we can also say about this study [12].

### Conclusion

Altogether, we can claim that this intervention was feasible and acceptable only for some groups of patients with specific educational and socioeconomic status meeting the requirements of the procedure, for example, access to and knowing how to use the internet. Through the last two decades, the opportunity to provide PE contents through internet-based platforms have been investigated. According to various studies, those platforms have shown good to acceptable retention rates [12-14,17,18,22,23]. However, all these earlier studies were designed and implemented in developed countries. In countries such as Iran, this area is still undeveloped and in its infancy; therefore, implementing these novel interventions should be followed cautiously. Results of the current study confirm that using a web-based PE platform is acceptable and feasible for a specific subgroup of patients, and we may need to revise the intervention to tailor it to the needs and features of other patient groups. However, further studies in a larger sample pool should be conducted to reach more conclusive results.

## Appendix

Multimedia Appendix 1 Example of a psychoeducational video clip which was embeded in website in the second week of the course.

## Abbreviations

BD: bipolar disorder
PE: psychoeducation
